# Editorial: Combining multiple non-invasive images and/or biochemical tests to predict prostate cancer aggressiveness

**DOI:** 10.3389/fonc.2023.1156649

**Published:** 2023-02-14

**Authors:** Rulon Mayer, Steven Raman, Charles B. Simone

**Affiliations:** ^1^ Department of Radiation Oncology, Perelman School of Medicine, University of Pennsylvania, Philadelphia, PA, United States; ^2^ Oncoscore, Garrett Park, MD, United States; ^3^ Department of Radiology, University of California, Los Angeles Health System, Los Angeles, CA, United States; ^4^ Department of Radiation Oncology, New York Proton Center, New York, NY, United States; ^5^ Department of Radiation Oncology, Memorial Sloan Kettering Cancer Center, New York, NY, United States

**Keywords:** multi-parametric MRI, prostate cancer, multivariable regression analyses, PI-RADS, prostate serum antigen, spatially registered images, nomogram

## Introduction

This Special Topics Issue in *Frontiers in Oncology*, Genitourinary Oncology compiles research articles that noninvasively assess prostate tumors through combining multiple disparate independent quantitative data. The contributions to the Special Topics discuss the performance of more common resources and methods that have been employed in the medical arena, such as biomarkers, clinical data, visual inspection of multi-parametric MRI (mpMRI), as well as adapting and applying novel approaches derived from other fields that quantitatively assess spatially registered multi-parametric MRI (SRMP-MRI).

## Background

“What’s past is prologue,” William Shakespeare, The Tempest

“The past is a stepping stone, not a mill stone,” Robert Plant

Quantifiable research endeavors can benefit from combining multiple independent pieces of information or variables (1–3) to describe or ascertain a given condition or predict an outcome. The sources of input information may be garnered from biomarkers, clinical factors, meta information, human intelligence, detectors and/or images. Having multiple input factors that supplement and/or complement each other without mere duplication improves the accuracy of the predicted outcome. To aid combining disparate data in the clinic, nomograms (4) have provided a graphical tool for computing the likelihood of an effect due to a number of input variables. Standard measures can establish the significance of the input information for evaluating or achieving a desired goal.

Extracting information, however, may burden and harm the patient (5). For prostate cancer, a 6-12 core transrectal ultrasound-based needle biopsy supplemented by MRI has been the principal means of diagnosis and patient risk stratification. Aside from possible under sampling the prostate (6, 7), such an invasive procedure carries the risks of pain, hemorrhage, and infection for the patient (8). Although the widely implemented non-invasive PSA indicator has significantly reduced PCa mortality, its low specificity lead to under and overtreatment and loss quality of life for the patient (9).

To improve PCa diagnosis, grading, and alleviate patient suffering, non-invasive strategies have been developed, such as the Prostate Imaging Reporting and Data System (PI-RADS) (9). PI-RADS (10) is a protocol for radiologists to visually inspect multiple MRI sequences and combine the assessments to determine the prostate tumor’s aggressiveness. However, such a qualitative approach depends on the training and experience of the radiologists.

Only one study (Jia et al.) in this compilation applies Artificial Intelligence (AI) methods to find image texture features and combine them to predict outcomes. AI harnesses the available image data and the growing computing power, is fashionable, and successful. However, there are drawbacks to the AI, such as overtraining of models lead to low accuracy, require fixed measurement conditions such as magnetic field, and textures are unconnected to physiology. The studies in this issue mostly avoid these pitfalls.

## Discussion

**Table 1** summarizes nine studies, including Chang et al., Falagario et al., Jia et al., Jiang et al., Lei et al., Liu et al., Mo et al., Wang et al., and Mayer et al. that examined the efficacy of combining various forms of PSA, prostate volume, and PI-RADS to non-invasively predict Clinically Significant Prostate Cancer (csPCa) or presence in MRI. There are a number of exceptions in this compilation. Chang et al. used two statistical metrics that characterize the diffusion, namely the mean and kurtosis to predict the International Society of Urological Pathology staging (ISUP). Falagario et al. added clinically based Risk Factors to mpMRI and improved the accuracy for detecting csPCa. Unlike other studies in this compilation, only Jia et al. applied AI and radiomics to predict the csPCa. Jiang et al. used geometric measures for the prostate to predict the presence of prostate cancer. Liu et al. departed from the others in examining input data that predicted the need for mpMRI. The summary cites the input variables, dependent variable, number of patients, an evaluation metric, specifically the Area Under the Curve (AUC) from Receiver Operator Characteristic and whether a nomogram was generated. All studies achieved high AUC and showed that adding mpMRI and using multiple variables relative to a single variable improved the accuracy. All studies need further verification with prospective studies and higher patient numbers.

**Table 1:** Retrospective, single center studies included in this Special Topics issue.

Author	Input Variables	Prediction	Number of Patients	Evaluation Metrics	Best or Range of AUC	Nomogram
Chang	D_mean_ ,D_kurtosis_	ISUP	45	AUC	0.907	No
Faligario	mpMRI, RC	csPCa	221	AUC, DCA	0.8	No
Jia	Radiomics: T2, DWI, Clinical Data	PFS	191	AUC, DCA, Calibration Curve	0.917-0.926	No
Jiang	Age, PSA, transCGA, PA	PCa	691	AUC	0.918	Yes
Lei	PI-RADS, PSAD	csPCa	422	AUC	0.97	No
Liu	Total PSA, Free PSA, PSAD, Prostate Volume, Age	Tumor Presence in MRI	784	AUC, DCA	0.8	No
Mo	Prostate Health Index (Free PSA, Total PSA), PI-RADS, Prostate Volume	csPCa	315	AUC	0.882	Yes
Wang	PI-RADS, PSAD	csPCa	833	AUC	0.94	No
Mayer	Eccentricity, Signal to Clutter Ratio, Tumor Volume	csPCa	25	AUC, DCA	0.861-0.969	Yes

mpMRI, Multi-parametric MRI; PI-RADS, Prostate Imaging Reporting And Data system; AUC, Area Under the Curve; DCA, Decision Curve Analysis; RC, Risk Calculator; PSAD, Prostate Serum Antigen Density; PFS, Progression Free survival; ISUP, International Society of Urological Pathology; D_mean_, Mean diffusion; D_kurtosis_, Diffusion kurtosis; csPCa, Clinically Significant Prostate Cancer; PA, maximum prostate sectional area; transCGA, transverse central gland area.

Two articles in this Special Topics issue studied spatially registered hyperspectral mpMRI. The first (Mayer et al.) eschewed the familiar independent variables (PI-RADS, PSA, age, etc), but instead tapped variables associated with SRMP-MRI such as eccentricity, Signal to Clutter Ration and achieved high AUC. The second (Mayer et al.), not in **Table 1**, does not use multiple features to predict an outcome. Instead, Mayer et al. studied an anomaly detector that finds deviant voxels within the normal prostate through processing the SRMP-MRI and examines a variety of statistical methods to manipulate the covariance matrix in order to generate an optimized AUC. Further studies are warranted that compared the anomaly detection with a radiologist tumor contouring of the SRMP-MRI tumors.

## Future research

“The best way to predict the future is to create it.” Abraham Lincoln

“The past is in your head, the future is in your hands.” Margaret Atwood

The works presented in this issue directly suggested future refinements, such as more patients, prospective studies, application to greater number of clinics, but hints at more ambitious projects such as:

### New biomarkers.

A number of studies in Special Topics showed that adding PSA to mpMRI boosts sensitivity and specificity for reliably determining csPCa. New biomarkers, beyond PSA (11, 12), show promise in identifying the presence of prostate tumors with fewer false positives than PSA. Future studies might combine these novel biomarkers with PI-RADS or mpMRI for further improvement.

### Directed proton therapy

Due to the increasing prevalence of proton beam therapy and its ability to more precisely deliver radiation therapy (13), imaging (14, 15) may reveal that certain patients benefit from exposing only a portion of the prostate, rather than the entire prostate, to irradiation, thus reducing possible side effects from unnecessarily exposing nearby normal tissues. To date, only treatment planning studies (13) suggest the feasibility of using mpMRI for this purpose.

### Qualitative/quantitative color maps.

Currently radiologists (10) visually inspect individual greyscale images to discern and interpret lesions. An alternative coloring schemes assigns red, green, blue to components in SRMP-MRI and generate a composite color image that can be quantified (16–18). Color in this case codes for PCa and normal tissue physiology. This coloring is not equivalent to false or pseudo coloring applied to individual images to show relative intensities within a given image. Future research (18) may clinically test employing tumor color display for patient care management and possibly derive new quantitative metrics for assessing tumors.

### Cross-clinic transformation

MRI scanning conditions (magnetic field strength, pulse sequences etc.), can vary among clinics which hinders AI-based techniques from generalization. Previously (19), “whitening-dewhitening” transformed target signatures for supervised target detections to handle the changes in conditions. Similarly (20), signatures based on Gleason score status were transformed. Future research may transform prostate tumor signatures across multiple clinics. A single library may hold multiple tumor signatures in the future.

### mpMRI and genomics

Other research directions may combine multiple data input or images to infer tumor genomics. A meta-analysis (21) found that mpMRI-visible cancer related to genotype, phenotype, physiology (proliferative signaling, DNA damage, and inflammatory processes). Others (22, 23) correlated mpMRI visibility with aggressive genomic and proteomic features. Further research incorporating all mpMRI modalities may further discriminate among genomic metrics or find more markers.

### Magnetic resonance spectroscopy

MRS uses many bands, similar to airborne hyperspectral imagers. However, MRS suffers from crude spatial resolution (MRS (24) is 0.25 cm^3^ versus mpMRI is 0.006 cm^3^) causing sampling issues. The limited MRS sample number precludes exploiting the statistical analysis due to background covariance matrix inversion non-singularity. Covariance matrix regularization can mitigate the insufficient sampling. Elevating the MRS spatial resolution by degrading the spectral resolution may enable MRS statistical analysis similar to remote sensing. Remote sensing proved the value of making the trade-offs and possibly help the clinic.

## Author contributions

Conception and design: RM, CS. Administrative support: RM, CS. Collection and assembly of data: RM. Data analysis and interpretation: RM. Writing of manuscript: RM, SR, CS. All authors (RM, SR, CS) listed have made a substantial, direct, and intellectual contribution to the work and approved it for publication.
